# Book Reviews

**Published:** 1892-06

**Authors:** 


### Book Reviews.

THE MEDITERREANIAN SHORES OF AMERICA, SOUTHERN CALIFORNIA, ETC.—Climatic, physical and meteorological conditions, by P. C. Remondeno, M. D., San Diego, Cal.

The introductory chapter is taken up in discussing climatology, the relation it bears to the etiology of tuberculosis. The following texts are successfully disposed of in an interesting style, viz.: Meteorological conditions and disease; sea air and marine climate; ocean moisture and soil moisture; rain and rainy weather on coasts; misapplied terms as to moist climate; extreme dryness of the air; consumption and temperature; geographical limits of consumption; the relative merit of altitude; why phthisis is more prevalent on lower elevations; are altitudes exempt from consumption? equability of temperature an important factor; different effects of sunshine and shade.

To sum up the writer's views briefly, he concludes that Southern California is all that can be desired as a home for the phthisical and invalids from other causes, that require an equitable and aseptic atmosphere. These conclusions are based upon observations and experience in this section of California covering a series of years devoted to the study and treatment of disease. The writer thinks that the tubercle bacilli will ultimately disappear in an atmosphere so unfavorable for its growth and well being. Glowing description is given of the beautiful and picturesque landscape and the salubrious and enchanting climate. The illustrations render the book attractive. The volume would form a valuable addition to the physician's library. It is published by the F. A. Davis Company, and is in keeping with the neatness and taste so characteristic of the company. A. B. P.

---

BACTERIOLOGICAL DIAGNOSIS.—Tabular aid for use in practical work. By James Eisenberg, Ph. D., M. D., Vienna. Translated by Norval H. Pierce, M. D. Published by the F. A. Davis Co.

This tabulated monogram is a valuable addition to the aids of bacteriological research to the practical worker. It is time saving in keeping before him the differential points in summing up a diagnosis of a species of micro-organisms. Koch's methods of analysing or dissecting (so to speak) bacteria are adhered to. The appreciation of bacteriologists of this book is shown in the fact that the first and second editions appeared within a year.

The appendix gives the technique used in the cultivation and staining of bacteria. A. B. P.

